# Efficacy of Spinosad Tablets Administered to a Colony of 15 Indoor Cats Naturally Infested with Fleas

**DOI:** 10.1155/2014/484308

**Published:** 2014-02-05

**Authors:** Marie-Christine Cadiergues, Charline Pressanti

**Affiliations:** INP-ENVT, 23 Chemin des Capelles, 31076 Toulouse Cedex 3, France

## Abstract

The aims of the study were (i) to describe adult fleas distribution in a strictly indoor cat colony composed of cats with flea allergy dermatitis (FAD) and non-FAD cats and (ii) to evaluate the efficacy of spinosad used alone. Skin lesions were scored according to the SCORing Feline Allergic Dermatitis lesion severity scale (SCORFAD) on days 0, 15, 30, 45, 60, and 90. Cats were combed prior to the treatment (days 0, 30, and 60) and on days 15, 45, and 90; collected fleas were replaced on the animals. All cats received flavored spinosad tablets (Comfortis) at a dosage of 50–75 mg/kg on days 0, 30, and 60. Cats were fed immediately afterwards. On day 0, a total of 60 fleas were collected (mean: 4 ± 4). Cats with FAD had a SCORFAD of 6, 8, 12, and 13 and harbored 0, 2, 1, and 0 fleas, respectively. Tablets were taken voluntarily by 8, 11, and 12 cats on days 0, 30, and 60, respectively. No adverse event was recorded. From day 15 to day 90, no fleas could be collected. SCORFAD was reduced by 40%, 71%, 80%, 89%, and 98% on days 15, 30, 45, 60, and 90, respectively.

## 1. Introduction

Fleas remain the most common parasites in cats [1–4]. In addition to potentially carry zoonotic diseases [5], fleas cause skin irritations due to their bites, including in some animals an allergic dermatitis (the so-called flea allergic dermatitis or FAD) [6, 7]. FAD is the most common allergic skin disease of dogs and cats, although its frequency varies according to geographical location. The past twenty years have brought important advances in flea biology as well as better insecticides [8]. Nevertheless, flea control in general, and more specifically in cats with FAD, remains a real challenge for veterinarians and owners. The goal is to minimize flea bites, that is, to minimize the amount of saliva injected by fleas in order to be under the allergic threshold.

Spinosad is an aerobic fermentation product of the soil bacterium, *Saccharopolyspora spinosa*. Spinosad kills insects through activation of the acetylcholine nervous system through nicotinic receptors. A chewable tablet is indicated for the treatment and prevention of flea infestations caused by *Ctenocephalides felis*; it was first introduced for dogs to the USA market in 2007 after approval by the Food and Drug Administration. The European Commission granted a marketing authorisation valid throughout the European Union in 2011 after approval by the European Medicines Agency. Subsequently, it was approved for cats in USA, Canada, and Japan in 2012 and in the European Union in 2013. Its excellent efficacy against fleas and its rapid killing effect that are observed in dogs [9–12] are also reported in cats [13, 14].

We had the opportunity to observe a colony of fifteen cats, housed strictly indoors and naturally infested with fleas; it included cats with clinical signs of FAD. We aimed to describe the distribution of fleas in a strictly indoor cat colony composed of FAD cats and non-FAD cats. We also aimed to evaluate the efficacy of spinosad used alone, both on the adult flea population and on the skin lesions.

## 2. Materials and Methods

Within a laboratory colony of fifteen strictly indoor, adult domestic shorthair cats, receiving no ectoparasiticide, four of them presented with excessive licking and alopecia, which had progressed over the past month. Advice was sought from the dermatology clinics of the Small Animal Hospital of the Toulouse Veterinary School. Clinical examination, test procedures, treatment, and followup of the cats were done after obtaining a written consent from the people responsible of the cat colony.

Coat brushing had allowed demonstration of flea infestation (adult fleas and/or flea dirt). *Ctenocephalides felis* was identified after microscopical examination of several adult specimens [15]. Skin scrapings and hair plucks had been negative for *Demodex* mites and dermatophytes. Tape impressions from the skin surface had not shown evidence of secondary bacterial or yeast infections.

Domestic shorthair cats (7 females spayed and 8 males neutered) between 4.7 and 6.7 years of age and weighing from 3 to 4.7 kg were housed in a stone-floored four-room space, measuring 23 m² and having an additional surface of 8 m² of cat tree furniture. No outdoor access was allowed. They had been living in this space for more than one year. They were fed a commercial cat diet and water was supplied ad libitum. Temperature was maintained at 19 ± 2°C and air was renewed 20 times per hour. Cats were vaccinated against feline panleukopenia virus, feline herpesvirus 1 (FHV-1), and feline calicivirus (FCV) and had no medical history.

Each animal was submitted to a full clinical examination on days 0, 15, 30, 45, 60, and 90. Skin lesions were scored according to the SCORing Feline Allergic Dermatitis lesion severity scale (SCORFAD) [16]. SCORFAD reduction was calculated at each time point *t* using the arithmetic mean of SCORFAD according to the following formula: SCORFAD  reduction  (%) = 100 × (mean_day  0_ − mean_*t*_)/mean_day  0_.

Cats were combed for 10 minutes prior to the treatment (days 0, 30, and 60) and additionally on days 15, 45, and 90. The combing procedure was standardized and applied similarly to every single cat. Two operators were involved in the assessment of a specific animal. One person handled and restrained gently the cat; the second combed the cat, quantified the fleas recovered from each comb, and recorded the data. During combing, an extra-fine flea comb (11.4 teeth/cm, http://www.easypets.fr) was used to recover fleas present in the cat's fur. The method of combing included several strokes of the comb in each area of the animal, each time moving in the same direction, following the pattern of the hair coat. Movement from one part of the cat's fur to the next was via strokes overlapping each other, so that no area of fur was missed. After completion of the combing procedure for all body areas, the whole procedure was repeated so that all areas were combed twice [17]. Fleas which were collected were counted and were replaced on the animal. Efficacy was calculated at each time point *t* using the arithmetic mean numbers of fleas according to the following formula: Efficacy  (%) = 100 × (mean_day  0_ − mean_*t*_)/mean_day  0_.

All cats received flavored spinosad tablets (Comfortis, Eli Lilly and Company Ltd., Basingstoke, UK) at a dosage of 50–75 mg/kg on days 0, 30, and 60. The tablet was first offered to the cat; in case of nonimmediate voluntary reception, the tablet was given with a pilling device. Cats were fed their usual ration immediately afterwards. All cats were observed for 2 hours posttreatment to record any adverse event. The environment was routinely cleaned, but no insecticide or insect growth regulator was applied. No inflammatory treatment was prescribed.

## 3. Results

**Figure 1:**
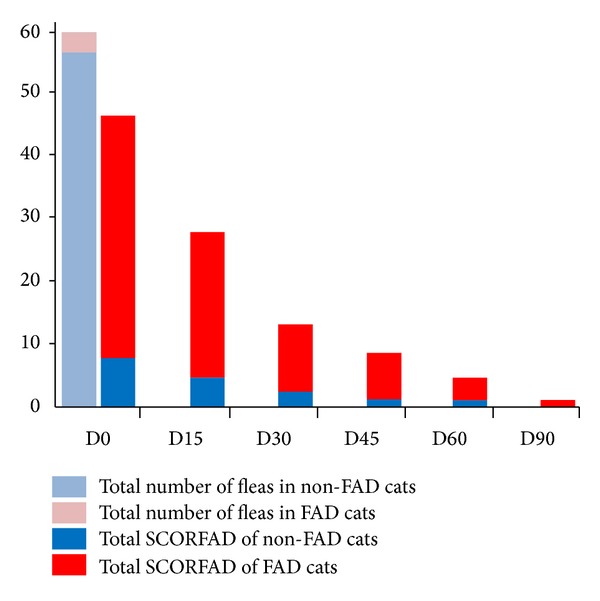
Progression of the total number of fleas (pale blue: non-FAD cats, pink: FAD cats) and the total SCORFAD (blue: non-FAD cats, red: FAD cats) over time.

On day 0, prior to the initial treatment, a total of 60 fleas were collected (mean: 4 ± 4, min: 0, max: 12). Two cats had self-induced alopecia, mainly on the dorsolumbar and dorsal tail regions. Two cats suffered from miliary dermatitis, principally of the dorsum and self-induced alopecia. The four cats with FAD had a SCORFAD of 6, 8, 12, and 13 and harbored 0, 2, 1, and 0 fleas, respectively. Additionally, very mild lesions were observed on five other cats (SCORFAD of 1 or 2) (Figure 1). Non-FAD cats harbored a total of 57 fleas (mean: 5.2 ± 4.1, min: 0, max: 12).

Tablets were taken immediately and voluntarily by 8, 11, and 12 cats on days 0, 30, and 60, respectively. No adverse event was recorded. From D15 to D90, no fleas could be collected (100% efficacy). SCORFAD was reduced by 40%, 71%, 80%, 89%, and 98% on days 15, 30, 45, 60, and 90, respectively (Figure 1).

## 4. Discussion

Flea infestation in strictly indoor animals, particularly cats, is frequently overlooked, possibly denied. In a study conducted in 2007 in UK, 48% of the owners whose pets had signs of an active flea infestation were unaware that their pet had fleas [1]. Cat owners may think that because they have an indoor-only cat, it is impossible for their pet to get fleas. Initially one or two fleas may be introduced from a pant leg, sock, or shoe after the owner had been gardening, walking, and so forth. In multipet households, a dog might be infested during walks. Then, ideal conditions for the life cycle of the cat flea (relative humidity of 70% and a temperature of between 20 and 30°C) are provided by a modern home environment. Furthermore, cats have such a nomadic sleeping behavior that bed underneath, sills, boxes, suitcases, planter boxes, and tops of furniture are but a few of the places that cats curl up and nap. All these places will constitute ideal places for flea life cycle. Most of the time, the infestation level will remain relatively low [2] due to the feline grooming behavior, making flea identification difficult, especially if there is a flea infestation denial. This was the case in the present situation with an average of 4 fleas per cat and caregivers who were convinced that flea infestation was not possible. Cats can be secretive and may not be observed grooming or traumatising themselves, increasing owner's disbelief.

In the present study, non-FAD animals harbored 5.2 fleas in average whereas FAD cats had only 0.75 fleas. Moreover, two cats had no fleas. Cats with FAD show commonly a very low level of infestation and sometimes do not have fleas at the time of the examination. These cats remove more fleas by grooming than do nonflea allergic cats since their level of pruritus is much higher. Therefore, physical evidence of fleas is reduced [18]. This confirms that FAD should not be ruled out based on the absence of fleas in a cat with skin lesions [19].

This clinical situation confirms the excellent efficacy and tolerance and the good palatability of spinosad tablets in cats [13, 14]. In the present study, no adverse event was observed, particularly no vomiting or diarrhea. Vomiting had been reported in 14% of the cats recruited in a large field trial [13]. Systemic treatment is well adapted to control fleas in animals with skin lesions. Excessive grooming, which is very common in cats with FAD, particularly in cases of self-induced alopecia, might reduce the amount of insecticide present on the skin thereby reducing or delaying its efficacy. Moreover, cutaneous inflammation and secondary cornification disorders (scaling) could impair the diffusion of topical products. Consequently, systemic products can be considered as the type of product of choice because the active ingredient is at a sufficient concentration despite overgrooming or a lesional skin. Spinosad acts very rapidly [11, 14], which is adequate in FAD animals: a fast-acting product decreases the total duration of the meals, thereby reducing the amount of saliva injected and consequently minimizing the allergenic stimulus [20]. Overall, 68.9% of the tablets were taken spontaneously when offered. The spontaneous acceptability was much higher than the data provided in the field study, where 11% of the tablets were taken spontaneously [13]. The cats included in the present study were particularly easy to medicate; this could explain the high level of spontaneous acceptability.

Clinical improvement took longer than ectoparasiticidal efficacy. Even after fleas have disappeared, skin inflammation persists, particularly when there are secondary infections or chronic pruritus. Part of the inflammation is self-induced, notably in cats with their tongues barbed. Furthermore, even if cats are no longer pruritic, hair regrowth takes time. In the current case, the two cats with miliary dermatitis lesions improved much more rapidly than the cats with self-induced alopecia. We elected not to use symptomatic relief as there was no secondary pyoderma and discomfort was considered as mild to moderate. FAD should not be ruled out before at least a two-month period of intense flea treatment [7], particularly in cats presenting with self-induced alopecia.

In households with several animals, it is essential to treat all animals, since the clinical unaffected animals ensure the environmental contamination with eggs. Within the colony of fifteen cats, 95% of fleas were collected from the non-FAD cats, who harbored in average 5 fleas whereas the FAD cats had less than 1 flea per cat in average. Cat flea egg production usually peaks during the night, coinciding with normal sleep periods for indoor pet dogs and cats [21]. As a consequence, there is a higher density of immature stages in animal resting places, including bedrooms.

We elected not to use any insecticide or insect growth regulator in the environment as the level of infestation was mild and external sources which could bring fleas were absent. It is usual to recommend flea control in indoor and outdoor environments in addition to treatments on pets [7, 8] to eliminate immature stages. However, modern products, with more rapid-killing effect and more persistent activity, applied on the pet only, may be sufficient to control flea infestation provided that all the animals are treated at the correct dosage and correct intervals. This is likely to be successful in a restricted environment (only-indoor pet) whereas indoor/outdoor pets are at higher risks of exposure to flea life stages present in the environment [19].

In conclusion, this clinical situation illustrated the fact that flea treatment should not be neglected in indoor animals. Cats with FAD were much less severely infested than cats without skin lesions; consequently, even in the absence of fleas FAD cannot be ruled out. Clinical improvement took three months despite the absence of adult fleas. This clinical situation also confirmed that spinosad given orally at a dose of 50–75 mg/kg was very well tolerated, easily accepted, and 100% effective against fleas in naturally infested adult cats.
